# Therapeutic Promise of Exosomal CD73 for Diabetic Bone Disorders: Addressing Mechanistic and Translational Challenges

**DOI:** 10.14336/AD.2025.1154

**Published:** 2026-09-12

**Authors:** Jing Lu, DuJiang Yang, Canhao Lai, Xiaoyu Chen, Ruichen Li, GuoYou Wang

**Affiliations:** ^1^The Affiliated Traditional Chinese Medicine Hospital of Southwest Medical University, Luzhou, Sichuan, China.; ^2^Center for Orthopedic Diseases Research, Southwest Medical University, Luzhou, China.

Dear Editor,

The recent study by Zhang et al. [1] offers compelling insights into the therapeutic potential of bone marrow mesenchymal stem cell (BMSC)-derived exosomal CD73 in diabetic bone disease. Specifically, the authors demonstrate how CD73 facilitates M2 macrophage polarization and enhances osteoblastic differentiation under diabetic conditions via the adenosine-A2bR-cAMP-PKA signaling pathway. These findings open a promising avenue for treating diabetes-associated skeletal disorders. Nonetheless, several mechanistic and translational considerations merit further exploration.

Zhang et al. convincingly show that CD73-deficient exosomes impair M2 polarization, whereas overexpression of CD73 reverses this defect. The use of a spontaneous type 2 diabetes model and adenosinergic inhibitors (e.g., PSB1115) adds physiological relevance. Their integration of in vivo and in vitro models provides a robust foundation for understanding exosomal CD73’s role in modulating immune responses in diabetic bone environments.

However, the complexity of adenosine receptor signaling warrants further elaboration. While A2aR (a high-affinity receptor) promotes M2 polarization and dampens pro-inflammatory cytokine release, A2bR—a low-affinity receptor upregulated in hyperglycemia or hypoxia—displays dual roles. It may mitigate inflammation or paradoxically enhance IL-6 production depending on context [4]. In diabetes, chronic hyperglycemia disrupts the fidelity of both receptors, complicating the net immunometabolic effect. Future studies should delineate A2aR and A2bR interactions using receptor-specific agonists/antagonists (e.g., ATL146e for A2aR; MRS1754 for A2bR) or cell-type-specific knockouts to resolve these dynamics within diabetic bone niches.

Moreover, diabetes alters exosomal content far beyond CD73, impacting miRNAs and proteins that may cooperatively modulate macrophage function. Multi-omics approaches—such as proteomics and RNA-seq comparing diabetic (Exo^dm) and healthy (Exo^wt) exosomes—could identify novel cargoes contributing to immune modulation. Additionally, while M2 polarization is emphasized, macrophage plasticity under chronic diabetic stress remains under-characterized. Tools like CX3CR1-based lineage tracing could reveal whether exosomal CD73 induces stable phenotypic shifts or transient modulation.

From a translational perspective, CD73-enriched exosomes are promising but face significant production and regulatory hurdles. These include low yield, contamination with apoptotic vesicles, batch variability, and instability during storage. Standard purification methods (e.g., ultracentrifugation or chromatography) often reduce functional recovery [5]. Furthermore, the lack of validated potency assays (e.g., specific surface markers or functional readouts) complicates reproducibility and quality control.

To address these challenges, synthetic exosome mimetics have emerged as promising alternatives. For example, bone-targeting exosome mimetics (EMs) functionalized with hydroxyapatite-binding ligands accumulate selectively in bone tissues and enhance regeneration in diabetic rodent models. EMs loaded with Smoothened agonist (SAG) have shown efficacy via Hedgehog pathway activation while minimizing off-target effects. Similarly, plant-derived exosome-like nanoparticles (PLNs), extracted from medicinal herbs, exhibit low immunogenicity, high stability, and bioactivity. In diabetic models, PLNs loaded with miRNA or immunomodulators effectively promoted M2 polarization and osteogenesis—offering scalable and biocompatible platforms for future therapy.

These innovative platforms not only overcome manufacturing limitations but also offer modularity for functionalization, improved targeting, and clinical translation in diabetic osteopathy.

We also propose a conceptual model positioning exosomal CD73 as a critical signaling node within the immune-bone-metabolism axis. In this model, CD73 delivered via BMSC-derived exosomes hydrolyzes extracellular ATP into adenosine within diabetic bone marrow niches. This adenosine activates macrophage A2aR or A2bR, driving M2 polarization and suppressing inflammation. The resulting immunoregulatory environment promotes osteogenesis. Thus, CD73 functions not merely as an enzyme but as a pivotal communicator that integrates metabolic stress (hyperglycemia), immune reprogramming (macrophage phenotype), and bone remodeling. Targeting this axis—whether via CD73-enriched exosomes or synthetic mimetics—could recalibrate dysregulated immunometabolism in diabetes and enhance bone healing.

In summary, Zhang et al. provide a strong mechanistic foundation for targeting exosomal CD73 in diabetic bone disease. Their work underscores the need for deeper mechanistic insight and translational innovation. Future studies should validate these findings in human diabetic tissues and explore combination strategies—such as immunomodulatory and osteoanabolic co-delivery—to advance bone regeneration in metabolic disease.
